# Drought stress-induced changes of microRNAs in diploid and autotetraploid *Paulownia tomentosa*

**DOI:** 10.1007/s13258-016-0473-8

**Published:** 2016-10-20

**Authors:** Xibing Cao, Guoqiang Fan, Lin Cao, Minjie Deng, Zhenli Zhao, Suyan Niu, Zhe Wang, Yuanlong Wang

**Affiliations:** 1 Institute of Paulownia, Henan Agricultural University, No 95 Wenhua Road, Jinshui Area, Zhengzhou, 450002 Henan People’s Republic of China; 2 College of Forestry, Henan Agricultural University, No 95 Wenhua Road, Jinshui Area, Zhengzhou, 450002 Henan People’s Republic of China

**Keywords:** *Paulownia tomentosa*, microRNA, Drought stress, Autotetraploid, Degradome analysis

## Abstract

Drought stress adversely affects plant productivity. Growth and timber production of Paulownia trees are limited under drought stress. Changes in gene expression patterns and miRNA in different ploidy of *Paulownia tomentosa* have been investigated. However, the responses of *P. tomentosa* to drought stress at the microRNA (miRNA) level have not been reported so far. To identify miRNA candidates and their target genes involved in the drought stress response in diploid and tetraploid *P. tomentosa*, four small RNA and four degradome libraries from diploid and autotetraploid *P. tomentosa* under normal and drought stress conditions were constructed and sequenced. A total of 41 conserved and 90 novel miRNAs were identified. Among these miRNAs, 67 (26 conserved and 41 novel) and 53 (six conserved and 47 novel) were significantly differentially expressed in response to drought stress in diploid and autotetraploid *P. tomentosa*, respectively. Degradome analysis identified 356 candidate miRNA target genes that encoded proteins with functions that included plant defense, transcriptional regulation, and hormone metabolism. In particular, miR4 and miR156 were identified only in autotetraploid *P. tomentosa* under drought stress. These results will help us build a foundation for future studies of the biological functions of miRNA-mediated gene regulation in *P. tomentosa*.

**Electronic supplementary material:**

The online version of this article (doi:10.1007/s13258-016-0473-8) contains supplementary material, which is available to authorized users.

## Introduction

Paulownia is a genus of fast-growing deciduous tree species in the family Paulowniaceae native to China. Paulownia trees are characterized by high biomass production and lightweight strong wood. The wood is used in house construction, farm implements, plywood, toys, musical instruments, and paper making. The trees are also used in environmental protection for the conservation of water and soil, wind prevention, and sand fixation (Ates et al. 2008; Ayan et al. 2006). Because the Paulownia root system can grow in deep soil, Paulownia is considered to be an eco-friendly multi-purpose species for use in agroforestry. Paulownia tree species have been successfully used in intercropping, and the plantation area was estimated to be over two million hectares (Lu 2006). Paulownia has become important to the Chinese economy, and is now a focus of research. In the past 10 years, our team has bred five autotetraploid Paulownia species from the corresponding diploids (Fan et al. 2007a, 2010, 2006, 2009, 2007b), which has greatly enriched the germplasm resources of Paulownia. However, drought is now recognized as an important factor that limits the growth and production of Paulownia. With the possible effects of global warming becoming a focus of attention, improving the tolerance of plants to drought has become a primary objective in plant breeding. Identifying drought resistance genes in Paulownia will help in understanding the mechanism of the Paulownia response to drought stress.

microRNAs (miRNAs) are an extensive class of endogenous noncoding small RNAs (sRNAs), which, as regulatory molecules, play important roles in plant growth and development, signal transduction, transcription regulation, and hormone metabolism under stress conditions(Candar-Cakir et al. 2016; Llave et al. 2002; Savi et al. 2016). Recently, the involvement of miRNAs in the response of *Paulownia fortunei* to salt stress has been reported (Fan et al. 2016). *Paulownia tomentosa* is a common tree in central China, and changes in miRNAs associated with ploidy have been reported in *P. tomentosa* (Fan et al. 2014). Until now, no studies have investigated the expression patterns of miRNAs and their roles in diploid and tetraploid *P. tomentosa* under drought stress. Therefore, in this study, we constructed four small RNA libraries from drought-treated and well-watered diploid and autotetraploid *P. tomentosa* seedlings to identify conserved and novel miRNAs, and also constructed four degradome libraries to predict the potential target genes of the detected miRNAs. We analyzed the differentially expressed miRNAs and their target genes in diploid and autotetraploid *P. tomentosa* under drought stress, which provided important insights into miRNA-mediated regulatory pathways and their putative target genes. The results will help in understanding the molecular mechanisms that underlie the response and adaption of *P. tomentosa* to water deficiency.

## Materials and methods

### Plant materials and dehydration stress

All the plant material was obtained from the Institute of Paulownia, Henan Agricultural University, China. Diploid and autotetraploid *P. tomentosa* tissue culture seedlings were first cultured for 30 days and then the plants were transferred into nutrition blocks containing ordinary garden soil. After 30 days, samples from diploid and autotetraploid *P. tomentosa* with the same height and crown size were transferred into plastic pots of 30-cm diameters with trays underneath containing ordinary garden soil for 50 days. Then, the seedlings were housed randomly in an outdoor nursery before being subjected to the drought stress treatment. Diploid and autotetraploid *P. tomentosa* with 75 % (control) and 25 % (drought stress) relative soil water contents were named PT2 and PT2T, and PT4 and PT4T, respectively. Based on our previous study (Dong et al. 2014), the drought treatment times were set as 0, 6, 9, and 12 days (wilting state). After the treatments, three individuals with consistent growth conditions were chosen from each group for leaf collection. Fully expanded leaves (the second pair of leaves from the apex) were collected from each plant in each group and pooled. The samples from the well-watered PT2 and PT4 plants were picked only after 12 days. After picking, the leaf samples were frozen immediately in liquid nitrogen and stored at −86 °C until used.

### Small RNA library construction and sequencing

Total RNA was extracted from the plant leaf material (PT2, PT2T, PT4, and PT4T) with Trizol reagent (Invitrogen, Carlsbad, CA) according to the manufacturer’s instructions. Four small RNA (sRNA) libraries were constructed using a Truseq™ Small RNA Preparation kit (Illumina, San Diego, CA). Briefly, 4 µg of total RNA was first ligated with 5′ and 3′ adapters. The ligation products were used for a reverse-transcription reaction to produce single-stranded cDNA, which served as a template for PCR amplification. The cDNA was purified using polyacrylamide gel electrophoresis, and the 140- to 160-bp fragments were selected to produce the libraries for cluster generation. The libraries were sequenced on a GAIIx platform (Illumina) following the manufacturer’s standard cBot and sequencing protocols at the Beijing Genomics Institute, Shenzhen, China.

### Identification of conserved and novel miRNAs

Low quality reads, adapters, and 5′ primer contaminants were removed from the raw reads of the four libraries to obtain the clean reads. The length distributions of the 18–30 nt clean reads were analyzed and mapped to the *P. tomentosa* UniGene database (Dong et al. 2014). Perfectly matched reads were mapped against the GeneBank (http://www.ncbi.nlm.nih.gov/) and the non-coding RNA (ncRNA) database (Release 10; http://rfam.sanger.ac.uk/)using the Blastall software (http://www.ncbi.nlm.nih.gov/staff/tao/URLAPI/blastall/) to identify rRNAs, scRNAs, snoRNAs, snRNAs, and tRNAs. After removing these sequences, the remaining sequences were mapped to the known plant miRNAs in miRBase database (Release 21.0; http://www.mirbase.org/) to identify known miRNAs, the mapped sequences with no more than two mismatches were identified as the conserved miRNAs, subsequently, the potential novel miRNAs were identified using MIREAP (http://sourceforge.net/projects/mireap/) and RNAfold (http://rna.tbi.univie.ac.at/cgi-bin/RNAfold.cgi) to fold flanking sequences and predict secondary structures and estimate the minimum free energy (MFE) among the remaining unmapped sequences. If a sequence had a Dicer cleavage site, a MFE below the cutoff, and conformed to other criteria described by Meyers and Zhu (2008), it was considered to be a candidate novel miRNA.

### Differential expression of miRNAs in the four *P. tomentosa* libraries

The differential expression profile analysis of the miRNAs was based on the relative abundance of each miRNA in each library. The expressions of the miRNAs in four libraries were firstly normalized to transcripts per million. The fold change and *p* value were calculated as describe by Audic and Claverie (1997), the significantly differential criterion were the fold changes ≥ 1 or ≤ −1 and P-values ≤ 0.05. The formulas as follows:

normalized expression = actual miRNA count/total count of clean reads * 1,000,000.

Fold change = log2 (normalized read counts in one library/normalized read counts in the other library).

*P*-values:

$$P\left( {x|y} \right) = \left( {\frac{{_{{\mathop N\nolimits_{2} }} }}{{\mathop N\nolimits_{1} }}} \right)\frac{{\left( {x + y} \right)!}}{{x!y!(1 + \frac{{\mathop N\nolimits_{2} }}{{\mathop N\nolimits_{1} }})^{(x + y + 1)} }}$$

$$C(y\; \le\; y\min |x) = \sum\limits_{{y = 0}}^{{y \le y\min }} {P(y|x)}$$

$$D(y \;\ge\; y\max |x) = \sum\limits_{{y \ge y\max }}^{\infty } {P(y|x)}$$

The N1 and N2 represent the total number of clean tags in PT2 and PT2T (or PT4 and PT4T), respectively. The x and y represent the number of miRNAs surveyed in PT2 and PT2T (or PT4 and PT4T), respectively. C and D can be regarded as the probability discrete distribution of the *P* value inspection.

### Identification of miRNA targets by degradome sequencing

Four degradome libraries were constructed for miRNA target identification as described previously (Addo-Quaye et al. 2008; German et al. 2008). Briefly, total RNA was extracted from the leaves of PT2, PT2T, PT4 and PT4T. Approximately 4 μg total RNA from each sample was used for polyadenylation according to the Oligotex mRNA mini kit (Qiagen, Shanghai, China). T4 RNA ligase (Takara, Dalian, China) was used to add a 5′RNA adapter to cleavage products that possessed a free 5′-phosphate at their 3′ termini. The ligated products were reverse transcribed using oligo (dT) and PCR enrichment, and then digested with the restriction enzyme *Mme*I (NEB, Ipswich, MA). A double-stranded DNA adapter was ligated to the digested products using T4 DNA ligase (NEB). The ligated products were purified and amplified with 20 PCR cycles with 94 °C for 30 s, 60 °C for 20 s, and 72 °C for 20 s. The PCR products were sequenced on an Illumina HiSeq™ 2000 system.

To evaluate the potential functions of the miRNA target genes, the sequences were searched against the GenBank Nr(ftp://ftp.ncbi.nih.gov/blast/db/nr), Nt (ftp://ftp.ncbi.nih.gov/blast/db/nt) and Swiss-Prot databases (http://www.ebi.ac.uk/swissprot) using BLASTX. Gene Ontology (GO) annotations (http://www.geneontology.org/) under the three categories (molecular function, cellular component, and biological process) were assigned to the target genes (Conesa et al. 2005; Du et al. 2010). An E-value threshold of less than 10^−5^ was used in the BLASTX searches.

### Verification of miRNAs and their potential targets by qRT-PCR

The expression of the miRNAs and their targets from PT2, PT2T, PT4, and PT4T were validated using qRT-PCR. Total RNAs were extracted from the diploid PT2, PT2T-6d, PT2T-9d, and PT2T, and the autotetraploid PT4, PT4T-6d, PT4T-9d, and PT4T leaves, respectively. Three biological replicates were performed. The qRT-PCR was performed following Chen et al. (2005) using a SuperScript III platinum SYBR Green one-step qRT-PCR kit (Invitrogen, Carlsbad, CA, USA) on a CFX96TM Real-Time PCR System (Bio-Rad, Hercules, CA) according to the manufacturer’s instructions. The PCR conditions were 50 °C for 3 min, 95 °C for 5 min, then 40 cycles at 95 °C for 15 s, 55 °C for 30 s, and 40 °C for 10 min. The primers for target genes were designed using Primer Premier 5 software (Premier Biosoft International, Palo Alto, CA, USA) based on NCBI and transcriptome sequences of diploid and autotetraploid of *P. tomentosa*, just as previously described by Fan et al. (2014), U6 rRNA (the validated of miRNAs) and 18S rRNA (the validated of targets) were chosen as an endogenous reference gene for normalization. All reactions were run in triplicate for each sample, 2^−ΔΔCT^ method was used to analyze relative changes in gene expression (Livak and Schmittgen 2000; Schefe et al. 2006). The ANOVA analyses were performed using SPSS 19.0 software (IBM Corp., Armonk, NY) The sequences of the primers and reference genes are shown in Tables S1 and S2.

## Results

### Deep sequencing of the sRNA libraries

**Fig. 1:**
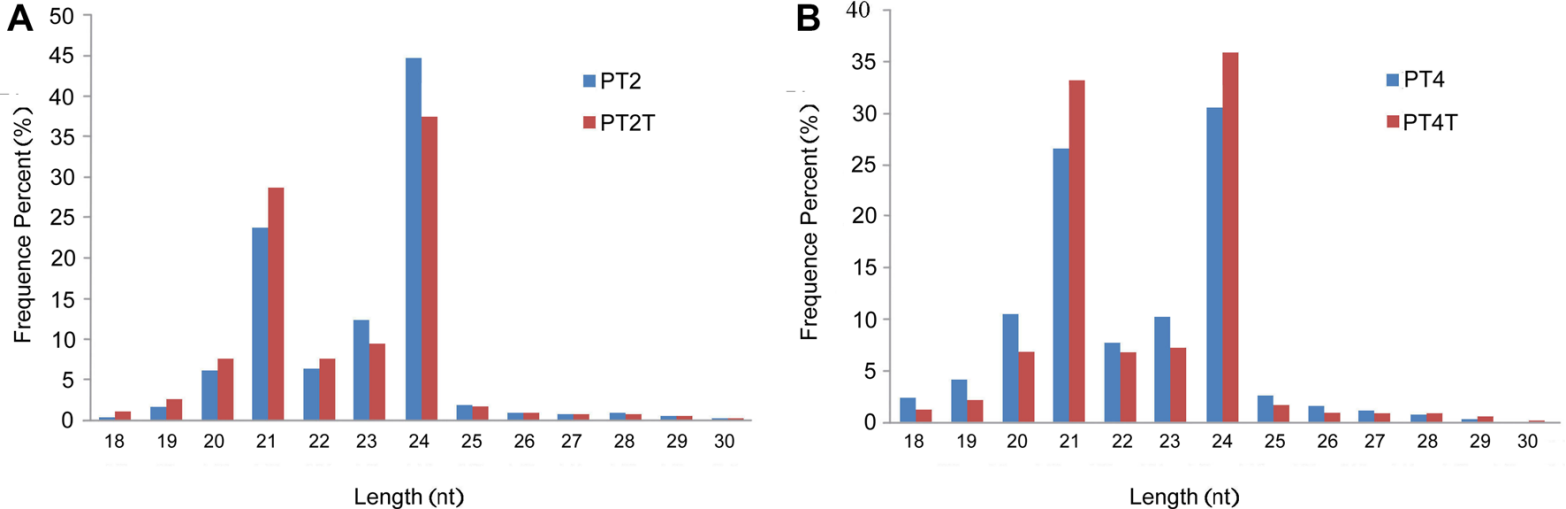
Length distribution of sRNAs libraries. **a** Size distribution of PT2 and PT2T libraries; **b** Size distribution of PT4 and PT4T libraries

A total of 12,772,899 (PT2), 17,316,751 (PT2T), 15,430,200 (PT4), and 15,486,302 (PT4T) raw reads were generated by high-throughput sequencing. After removing reads with poly(A) tails, low-quality reads, contaminated reads, and adaptor sequences, a total of 12,717,314 (PT2), 16,987,504 (PT2T), 15,110,539 (PT4), and 15,206,670 (PT4T) clean reads were obtained. The length distributions of the most abundant reads (18–30 nt) between two comparisons (PT2 vs. PT2T and PT4 vs. PT4T) are shown in Fig. 1. The 24-nt long miRNAs were the most abundant, which is consistent with the abundances reported previously in Nicotiana species and in *Solanum linnaeanum* (Yin et al. 2014; Zhuang et al. 2013). A total of 30 % of the sRNA reads were mapped to the Paulownia UniGene database (Dong et al. 2014) and the aligned sequences were classified and annotated as non-coding RNAs and miRNAs (Table 1) using Blastall searches against the GenBank, Rfam, and miRBase databases. The remaining unmapped sequences were used to predict potentially novel miRNAs.

**Table 1:** General statistics of sRNAs in *P. tomentosa*

	Category	Total	miRNA	rRNA	snRNA	snoRNA	tRNA	Unannote
PT2	Unique sRNAs	3991595	13530	47923	1206	449	10115	3918372
	Percent (%)	100	0.34	1.2	0.03	0.01	0.25	98.17
	Total sRNAs	12717314	2123920	391391	2037	939	413341	9785686
	Percent (%)	100	16.7	3.08	0.02	0.01	3.25	76.95
PT2T	Unique sRNAs	4515330	22248	58347	1809	656	14895	4417375
	Percent (%)	100	0.49	1.29	0.04	0.01	0.33	97.83
	Total sRNAs	16987504	3750931	593427	5324	1187	692398	11944237
	Percent (%)	100	22.08	3.49	0.03	0.01	4.08	70.31
PT4	Unique sRNAs	3749155	17053	90808	2211	787	18043	3620253
	Percent (%)	100	0.45	2.42	0.06	0.02	0.48	96.56
	Total sRNAs	15110539	2702653	1556856	5301	1688	1194746	9649295
	Percent (%)	100	17.89	10.3	0.04	0.01	7.91	63.86
PT4T	Unique sRNAs	3993845	17988	60787	2244	681	14627	3897518
	Percent (%)	100	0.45	1.52	0.06	0.02	0.37	97.59
	Total sRNAs	15206670	3663160	605528	6749	1116	409239	10520878
	Percent (%)	100	24.09	3.98	0.04	0.01	2.69	69.19

### Identification of conserved miRNAs in *P. tomentosa*

A total of 41 conserved miRNAs belonging to 18 miRNA families were identified (Table S3), Among these conserved miRNAs, miR5021 was the largest families with 13 members, followed by miR169 and miR482, both have three members. The miR166 family had two members but more than 930,000 reads in each of the four libraries, making it the most common family in the four libraries. miRNAs that were diploid or autotetraploid specific were also detected. For example, two conserved miRNAs (miR398 and miR171) were found only in PT2 vs. PT2T, and two conserved miRNAs (miR5021and miR482b) were found only in PT4 vs. PT4T. In addition, the drought-stressed PT2T and PT4T plants had very high expression levels of pau-miR2911, pau-miR166a, pau-miR166b, pau-miR167b, and pau-miR157 compared with the PT2 and PT4 controls. Interestingly all the conserved miRNAs except pau-miR408a, pau-miR408b, pau-miR168, and pau-miR390 were up-regulated by varying degrees in PT2T and PT4T compared with PT2 and PT4. These results show that different miRNAs had varied expression patterns in the four libraries.

### Identification of novel miRNAs in *P. tomentosa*

To identify candidate novel miRNAs, the secondary structures of the pre-miRNA sequences of the unmapped miRNAs were predicted using MIREAP. Ninety potentially novel miRNAs were identified among the four libraries, including 21 miRNAs with the corresponding miRNA* sequence (Table S4). The lengths of the novel mature miRNA sequences varied from 20nt to 23nt; the 21nt sequences were the most abundant. The average minimal folding free energy was −44.3 kcal/mol and the average length of the pre-miRNA was 143nt. Of the 90 novel miRNAs, 19 (21 %) were found in all four libraries, and 20 and 25 novel miRNAs were found in the PT2 vs. PT2T, and PT4 vs. PT4T libraries, respectively. The numbers of novel miRNAs found in each library are shown in Table S4.

### Analysis of differentially expressed miRNAs in diploid and tetraploid *P. tomentosa* under drought stress

Differentially expressed miRNAs with p-values < 0.05 and log2ratios > 1 were defined as significantly up-regulated and miRNAs with p-value < 0.05 and log2 ratio < −1 were defined as significantly down-regulated.

The differentially expressed miRNAs were both up-regulated or down-regulated in the two comparisions (PT2 vs. PT2T and PT4 vs. PT4T) were drought specific miRNAs. Compared with PT2, in PT2T, 41 miRNAs were significantly up-regulated and 26 miRNAs were significantly down-regulated. Compared with PT4, in PT4T, 32 miRNAs were significantly up-regulated and 21 miRNAs were significantly down-regulated (Table S3 and Table S4). In both PT2 vs. PT2T and PT4 vs. PT4T comparisons, six miRNAs including pau-miR156, pau-miR2911, pau-miR396a, pau-miR399, pau-miR160, and pau-miR43 were significantly up-regulated, and eight miRNAs, such as pau-miR1, pau-miR4, pau-miR5a, pau-miR5b, pau-miR17a, pau-miR17b, pau-miR21, and pau-miR27 were significantly down-regulated. Furthermore, four miRNAs, pau-miR3, pau-miR19, pau-miR56a, and pau-miR56b, were significantly down-regulated in PT2 vs. PT2T and up-regulated in PT4 vs. PT4T. These results suggest that significantly differentially expressed miRNAs may have important roles in the drought stress response of *P. tomentosa*.

### Degradome sequencing analysis of miRNA targets

**Fig. 2:**
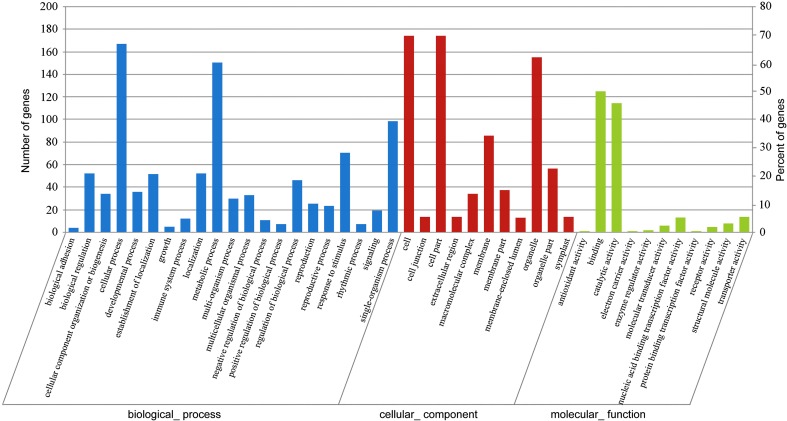
GO analysis of *P. tomentosa* miRNA targets

In order to identify the targets of the miRNA and predict their functions, we performed a degradome sequencing analysis (Han et al. 2016; Wei et al. 2013). A total of 356 targets and 773 cleavage sites were identified. The relative abundances of the miRNA targets were used to assign the transcripts to three categories (Addo-Quaye et al. 2008; German et al. 2008): category I with 121 (125 cleavage sites) targets, category II with 209 (594 cleavage sites) targets, and category III with 22 (54 cleavage sites) targets (Table S5). These results suggested that the targets could be cleaved efficiently by the miRNAs. To further understand the function of the targets, GO term was used to classify the function of these 356 targets. the results showed that 252 were annotated with 43 terms main including cellular process, metabolic process, cell, cell part, organelle, binding, and catalytic activity (Fig. 2).

### Expression pattern analysis of miRNAs and their targets with qRT-PCR

**Fig. 3:**
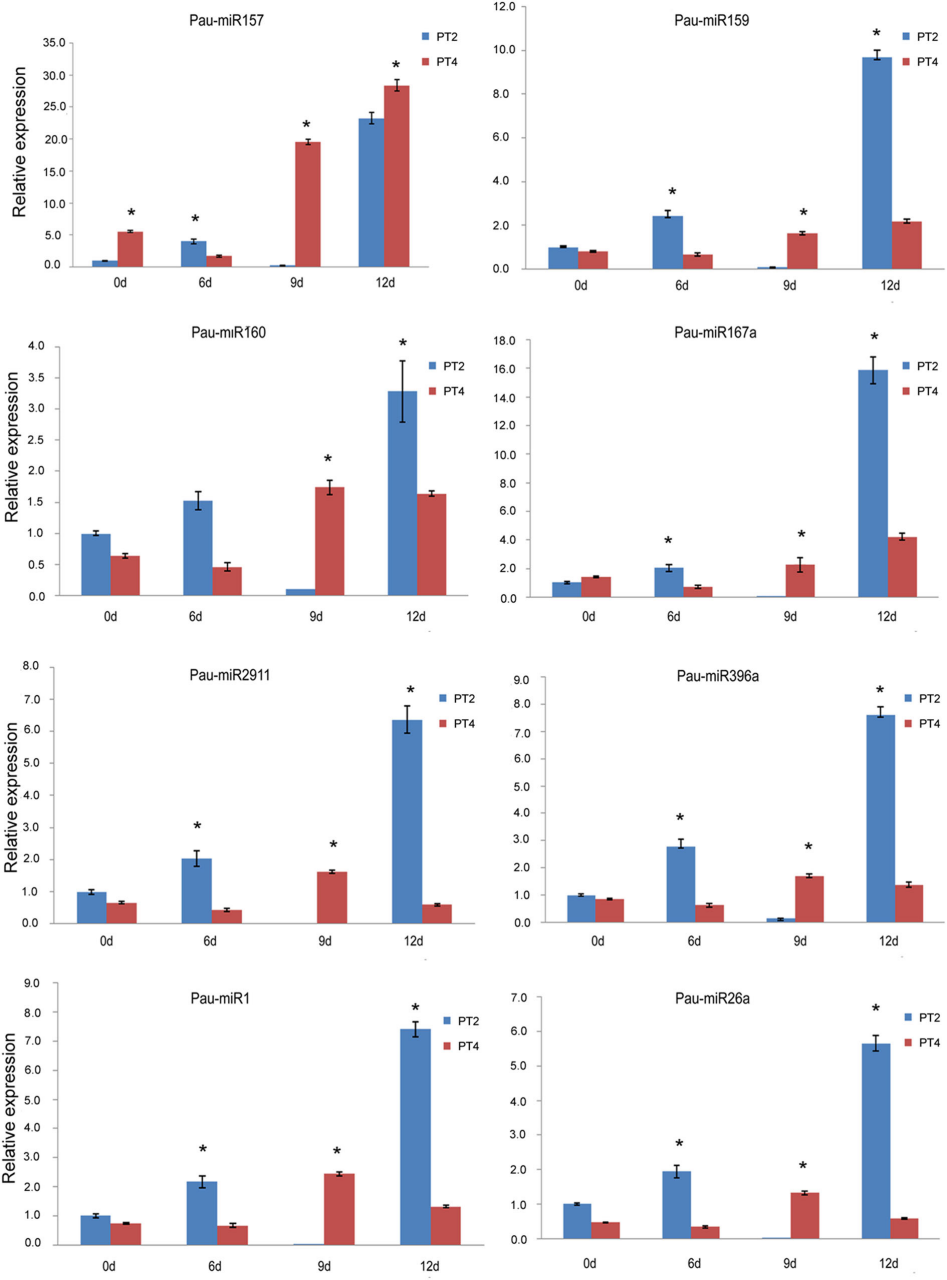
The qRT-PCR validation of the expression of miRNAs at different treatment stages

**Fig. 4:**
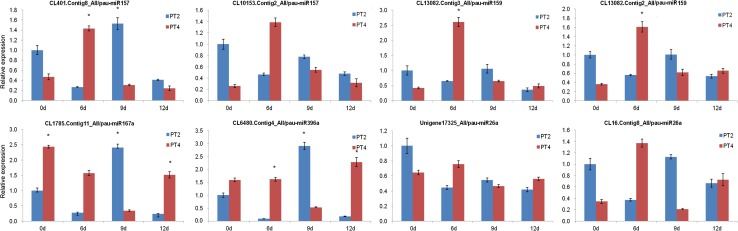
The qRT-PCR validation of the relative expression of target gene

To detect the miRNA expression level at different drought treatment time, eight of the differentially expressed miRNAs were selected randomly for validation by qRT-PCR. As shown in Fig. 3, the result showed that the trends of five miRNA expression (pau-miR396a, pau-miR159, pau-miR167a, pau-miR160, and pau-miR26a) were similar with high-throughput sequencing data. The expression levels of these five miRNAs in the diploid and tetraploid plants were up-regulated at day 6 of drought treatment, down-regulated at day 9, and up-regulated at day 12. The expression patterns of pau-miR2911, pau-miR157, and pau-miR1 showed similar trends between diploid and tetraploid plants at day 6 and day 9 of drought treatment. To confirm the reliability of degradome sequencing analysis and investigate the potential correlation between miRNAs and their targets, eight genes were selected for qRT-PCR assays (Fig. 4). The expression patterns of three of the targets (CL401.Contig8_All, CL10153.Contig2_All, and CL6480.Contig 4_All) were inversely correlated with the expression patterns of the corresponding miRNAs. The expression levels of CL13082.Contig3_All, CL13082.Contig2_All, CL1785. Contig11_All, CL16. Contig8_All, and Unigene17325_All were positively correlated with the expression levels of the corresponding miRNAs. These results show that, in *P. tomentosa* under drought stress, the expression patterns of the differentially expressed miRNAs and their targets were complex and varied.

## Discussion

Drought is a major abiotic stress factor that significantly reduces Paulownia production. In this study, we constructed and sequenced four *P. tomentosa* sRNA libraries and compared changes in the expression patterns of miRNAs in diploid and tetraploid *P. tomentosa* under drought stress. We detected a total of 120 differentially expressed miRNAs; 67 in PT2 vs. PT2T and 53 in PT4 vs. PT4T. To gain insights into the miRNA regulatory networks that may be involved in the drought stress response, we performed degradome sequencing to predict the targets of the miRNAs, and then the function of these miRNA-target were analyzed.

### miRNA–mediated gene expression regulation associated with drought resistance

Plant roots play important roles in plant growth and development through the uptake of water and nutrients from the soil, and roots are exposed directly to abiotic and biotic stresses. Auxin is an important phytohormone that controls many of the cellular processes during root apical meristem cell differentiation and elongation (Benková and Hejátko 2009; Khan et al. 2011). We identified the auxin response factor gene *ARF6*, which was predicted to be targeted by pau-miR167 (Table S5), ARF6 has been reported to play significant roles in plant root development (Khan et al. 2011; Meng et al. 2010). Pau-miR167 was up-regulated in PT2 vs. PT2T, whereas it was not detected in PT4 vs. PT4T. According to the negative regulation relation of miRNAs and their targets, *ARF6* will be down-regulated in PT2 vs. PT2T. In *Arabidopsis thaliana*, the low expression of *ARF* was found to result in defective root cap development (Wang et al. 2005), which reduced the water absorption ability of the roots and resulted in the withering of leaves under drought stress.

Previous studies have shown that the miR156 family is highly conserved and highly expressed in almost all plant species(Chen 2009; Xie et al. 2013). In this study, we found that pau-miR156 was significantly up-regulated in PT2 vs. PT2T and PT4 vs. PT4T. The predicted target gene of miR156 was squamosa promoter-binding-like protein 12 (*SPL12*), which encodes a plant-specific transcription factor that was reported to play crucial roles in promoting the transition from juvenile to adult growth, shoot maturation, leaf development, and flowering (Chuck et al. 2008; Cui et al. 2014), so the morphology and physiological changes in paulownia under drought stress may be associated with the negative regulation of miR156 (Dong et al. 2014). MiR159 family members have been shown to regulate plant MYB transcription factors. In this study, the predicted target gene of pau-miR159 was *GAMYB*. Pau-miR159 was significantly up-regulated in PT2 vs. PT2T, MYBs have been reported to regulate stomatal regulation under drought conditions, and their over-expression was found to result in hypersensitivity to water deficiency (Oh et al. 2011). According to the negative regulation of miRNAs with their targets, the expression levels of MYBs may have promoted guard cell deflation in response to drought stress, resulting in stomatal closure and limiting the growth the root of Paulownia, which further affect the water uptake ability of root. Together these results suggest that miR156–*SPL12*, pau-miR167–*ARF*, and pau-miR159–*GAMYB* regulation may be closely related to *P.tomentosa* drought tolerance.

### Drought induced miRNA–target regulation involved in cell signaling transduction

In this study, we indentified several miRNA targets involved in cell signaling transduction, such as zinc finger protein and zinc finger CCC-type protein, were the predicted targets for pau-miR4 (Table S5), which was down-regulated in both PT2 vs. PT2T and PT4 vs. PT4T. Zinc finger proteins are DNA-binding transcription factors that can enhance biotic and abiotic stress tolerance during plant growth and development. Other pau-miR4 targets were F-box proteins, which have been reported to play important regulatory roles in cell signaling (Kipreos and Pagano 1999). ACC oxidase, another one of the targets of pau-miR4, catalyzes the final step in the biosynthesis of ethylene, which is a signal for slowing growth in response to external stresses. Therefore, the low expression of pau-miR4 might enhance the activity of ACC oxidase so as to produce the ethylene required to slow plant growth and increase drought tolerance. The trend of the expression pattern of the ACC oxidase gene is similar to that reported previously (Dong et al. 2014). Pau-miR4 was also predicted to target alcohol dehydrogenase class-3-like isoform 1, which may be involved in drought stress signaling (Ranjan et al. 2012). Pau-miR4–ethylene-responsive transcription factor 12 and pau-miR4–gibberellin signaling DELLA protein regulation have also been related to plant hormone signaling associated with drought stress. The serine/threonine -protein kinase gene *NAK* was also predicted to be targeted by pau-miR4. *NAK* is an enzyme in the jasmonic acid signaling pathway (Piot 2003). Pau-miR159, which was up-regulated in PT2 vs. PT2T and did not change in PT4 vs. PT4T, was predicted to target *GAMYB*. This gene family is the largest transcription factor family in plants, and is a component of gibberellin signaling, The differential expression of *GAMYB* was reported to influence the promoter activity of the most predominant gibberellin-responsive gene *RAmy1A* in the gibberellin signaling pathway (Washio and Washio 2014). These results suggest that drought stress responses are very complicated is controlled by a various signaling net,and the co-ordination of pau-miR4–target and pau-miR159–target regulation might promote drought tolerance in *P. tomentosa*.

### Drought-specific miRNAs of *P. tomentosa*

To identify candidate drought-specific miRNAs of *P. tomentosa* from the differentially expressed miRNAs, we compared them with the miRNAs reported in a previous study in the same species. In the previous report, thirteen autotetraploid-specific miRNAs of *P. tomentosa* were identified, including miR4, miR156, miR160, miR166, miR169, miR171, miR396, miR397, miR398, miR399, miR408, miR482, and miR 858, which were strongly express in the PT2 vs. PT4 (Fan et al. 2014). In this study, drought-specific miRNAs of *P. tomentosa* were identified in the PT2 vs. PT2T and PT4 vs. PT4T comparisons, including pau-miR1, pau-miR4, pau-miR5a/b, miR156, miR160, miR396, miR399, and miR2911. Among them, the expressions of miR156, miR160, miR396, miR399, and miR2911 were up-regulated and the expression of pau-miR1, pau-miR4, and pau-miR5a/b were down-regulated in both diploid and autotetraploid *P. tomentosa* under drought stress. When we compared the expression levels of these miRNA in this and the previous study, only the expression trends of miR4, miR156, miR160, and miR399 were consistent in both studies. Thus, we propose that the differential expression patterns of these four miRNAs might be related to the different drought treatment conditions. Through degradome sequencing, only the target of miR4 and miR156 were mapped to the reference sequences, therefore, we speculate that miR4 and miR156 are drought-specific miRNAs of autotetraploid *P. tomentosa*. This important information will help to understand the drought response in Paulownia species and may be useful for the development of tolerant varieties in the future.

## Conclusions

In this study, we detected 67 and 53 significant differentially expressed miRNAs in response to drought stress in diploid and autotetraploid *P. tomentosa*, respectively. Eight of the miRNAs and eight of their targets were confirmed by qRT-PCR. Degradome sequencing analysis showed that miR4–target and miR156–target regulation could be provide valuable information in the drought stress response in diploid and tetraploid *P. tomentosa*. These results will help us build a foundation for further studies of the biological functions of miRNA-mediated gene regulation in *P. tomentosa* under drought stress.

## Electronic supplementary material

Below is the link to the electronic supplementary material.

Supplementary material 1 (DOCX 12 kb)

Supplementary material 2 (DOCX 13 kb)

Supplementary material 3 (XLSX 18 kb)

Supplementary material 4 (XLSX 29 kb)

Supplementary material 5 (XLSX 49 kb)

## References

[CR1] Addo-Quaye C, Eshoo TW, Bartel DP, Axtell MJ (2008). Endogenous siRNA and miRNA targets identified by sequencing of the Arabidopsis degradome. Curr Biol.

[CR2] Ates S, Ni Y, Akgul M, Tozluoglu A (2008). Characterization and evaluation of *Paulownia elongota* as a raw material for paper production. Afr J Biotechnol.

[CR3] Audic S, Claverie JM (1997). The significance of digital gene expression profiles. Genome Res.

[CR4] Ayan S, Sivacioglu A, Bilir N (2006). Growth variation of *Paulownia* Sieb. and Zucc. species and origins at the nursery stage in Kastamonu-Turkey. J Environ Biol.

[CR5] Benková E, Hejátko J (2009). Hormone interactions at the root apical meristem. Plant Mol Biol.

[CR6] Candar-Cakir B, Arican E, Zhang B (2016). Small RNA and degradome deep sequencing reveals drought-and tissue-specific micrornas and their important roles in drought-sensitive and drought-tolerant tomato genotypes. Plant Biotechnol J.

[CR7] Chen X (2009). Small RNAs and their roles in plant development. Annu Rev Cell Dev Biol.

[CR8] Chen C, Ridzon DA, Broomer AJ, Zhou Z, Lee DH, Nguyen JT, Barbisin M, Xu NL, Mahuvakar VR, Andersen MR, Lao KQ, Livak KJ, Guegler KJ (2005). Real-time quantification of microRNAs by stem-loop RT-PCR. Nucleic Acids Res.

[CR9] Chuck G, Candela H, Hake S (2008). Big impacts by small RNAs in plant development. Curr Opin Plant Biol.

[CR10] Conesa A, Götz S, García-Gómez JM, Terol J, Talón M, Robles M (2005). Blast2GO: a universal tool for annotation, visualization and analysis in functional genomics research. Bioinformatics.

[CR11] Cui LG, Shan JX, Shi M, Gao JP, Lin HX (2014). The miR156-SPL9-DFR pathway coordinates the relationship between development and abiotic stress tolerance in plants. Plant J..

[CR12] Dong Y, Fan G, Deng M, Xu E, Zhao Z (2014). Genome-wide expression profiling of the transcriptomes of four *Paulownia tomentosa* accessions in response to drought. Genomics.

[CR13] Du Z, Zhou X, Ling Y, Zhang Z, Su Z (2010). AgriGO: a GO analysis toolkit for the agricultural community. Nucleic Acids Res.

[CR14] Fan GQ, Yang Z, Cao Y, Liu F, Jia F (2006). Autotetraploid induction of *Paulownia elongata* with colchione. Acta Agr Nucleatae Sin.

[CR15] Fan GQ, Cao Y, Zhao Z, Yang Z (2007). Induction of autotetraploid of *Paulownia fortunei*. Sci Silv Sin.

[CR16] Fan GQ, Yang ZQ, Cao YC, Zhai XQ (2007). Induction of autotetraploid of *Paulownia tomentosa* (Thunb.). Steud Plant Physiol Commun.

[CR17] Fan GQ, Wei ZZ, Yang ZQ (2009). Induction of autotetraploid of *Paulownia australis* and its in vitro plantlet regeneration. J Northwest A & F Univ.

[CR18] Fan GQ, Zhai X, Wei Z, Yang Z (2010). Induction of autotetraploid from the somatic cell of *Paulownia tomentosa* × *Paulownia fortunei* and its in vitro plantlet regeneration. J Northeast Forest Univ.

[CR19] Fan GQ, Zhai X, Niu S, Ren Y (2014). Dynamic expression of novel and conserved microRNAs and their targets in diploid and tetraploid of *Paulownia tomentosa*. Biochimie.

[CR20] Fan GQ, Li X, Deng M, Zhao Z, Lu Y (2016). Comparative Analysis and Identification of miRNAs and their target genes responsive to salt stress in diploid and tetraploid *Paulownia fortunei* seedlings. PLoS ONE.

[CR21] German MA, Pillay M, Jeong DH, Hetawal A, Luo S, Janardhanan P, Kannan V, Rymarquis LA, Nobuta K, German R, De Paoli E, Lu C, Schroth G, Meyers BC, Green PJ (2008). Global identification of microRNA-target RNA pairs by parallel analysis of RNA ends. Nat Biotechnol.

[CR22] Han X, Yin H, Song X, Zhang Y, Liu M, Sang J, Jiang J, Li J, Zhuo R (2016). Integration of small RNAs, degradome and transcriptome sequencing in hyperaccumulator *Sedum alfredii* uncovers a complex regulatory network and provides insights into cadmium phytoremediation. Plant Biotechnol J.

[CR23] Khan GA, Declerck M, Sorin C, Hartmann C, Crespi M, Lelandais-Brière C (2011). microRNAs as regulators of root development and architecture. Plant Mol Biol.

[CR24] Kipreos ET, Pagano M (1999). The F-box protein family. Genome Biol.

[CR25] Livak KJ, Schmittgen TD (2000). Analysis of relative gene expression data using real-time quantitative PCR and the 2^−ΔΔCT^ method. Methods.

[CR26] Llave C, Xie Z, Kasschau KD, Carrington JC (2002). Cleavage of scarecrow-like mRNA targets directed by a class of Arabidopsis miRNA. Science.

[CR27] Lu J (2006). Energy balance and economic benefits of two agroforestry systems in northern and southern China. Agr Ecosyst Environ.

[CR28] Meng Y, Ma X, Chen D, Ping W, Ming C (2010). MicroRNA-mediated signaling involved in plant root development. Biochem Bioph Res Commun.

[CR29] Meyers BC, Zhu JK (2008). Criteria for annotation of plant microRNAs. Plant cell.

[CR30] Oh JE, Kwon Y, Kim JH, Noh H, Hong SW, Lee H (2011). A dual role for MYB60 in stomatal regulation and root growth of *Arabidopsis thaliana* under drought stress. Plant Mol Biol.

[CR31] Piot F (2003) Pattern of expression of the F8A24.12 gene from Arabidopsis thaliana which encodes a NAK ser/thr protein kinase and functional analysis of the suORF present in its 5′UTR. Chemistry & Biochemistry

[CR32] Ranjan A, Pandey N, Lakhwani D, Dubey NK, Pathre UV, Sawant SV (2012). Comparative transcriptomic analysis of roots of contrasting *Gossypium herbaceum* genotypes revealing adaptation to drought. BMC Genom.

[CR33] Savi T, Marin M, Luglio J, Petruzzellis F, Mayr S, Nardini A (2016) Leaf hydraulic vulnerability protects stem functionality under drought stress in *Salvia officinalis*. Funct Plant Biol 10.1071/FP15324 32480468

[CR34] Schefe J, Lehmann K, Ir Unger T, Funke-Kaiser H (2006). Quantitative real-time RT-PCR data analysis: current concepts and the novel “gene expression’s CT difference” formula. J Mol Med.

[CR35] Wang JW, Wang LJ, Mao YB, Cai WJ, Xue HW, Chen XY (2005). Control of root cap formation by MicroRNA-targeted auxin response factors in Arabidopsis. Plant cell.

[CR36] Washio K, Washio K (2014). Functional Dissections between GAMYB and Dof transcription factors suggest a role for protein-protein associations in the gibberellin-mediated expression of the *RAmy1A* gene in the rice aleurone. Genet Mol Res.

[CR37] Wei M, Wei H, Wu M, Song M, Zhang J, Yu J, Fan S, Yu S (2013). Comparative expression profiling of miRNA during anther development in genetic male sterile and wild type cotton. BMC Plant Biol.

[CR38] Xie F, Stewart CNS, Taki FA, He Q, Liu H, Zhang B (2013). High-throughput deep sequencing shows that microRNAs play important roles in switchgrass responses to drought and salinity stress. Plant Biotechnol J.

[CR39] Yin F, Gao J, Liu M, Qin C, Zhang W, Yang A, Xia M, Zhang Z, Shen Y, Lin H, Luo C, Pan G (2014). Genome-wide analysis of water-stress-responsive microRNA expression profile in tobacco roots Funct Integ Genomic.

[CR40] Zhuang Y, Zhou XH, Liu J (2013). Conserved miRNAs and their response to salt stress in wild eggplant Solanum linnaeanum roots. Int J Mol Sci.

