# Supplementary material for: Drought stress-induced changes of microRNAs in diploid and autotetraploid Paulownia tomentosa
Source: Genes Genomics. 2016 Oct 20;39(1):77–86. doi: 10.1007/s13258-016-0473-8 (PMC5196014; doi:10.1007/s13258-016-0473-8)
Supplement: Supplementary file 1 — Supplementary material 1 (DOCX 12 kb) [file 13258_2016_473_MOESM1_ESM.docx]

Table S1 Primers of miRNAs used for qRT-PCR

| Name | Sequence (5'-3') |
| --- | --- |
| Pau-miR2911 | GCCGGCCGGGGGACGGACTGGG |
| Pau-miR396a | TTCCACAGCTTTCTTGAACTG |
| Pau-miR159 | TTTGGATTGAAGGGAGCTCTA |
| Pau-miR167a | TGAAGCTGCCAGCATGATCTA |
| Pau-miR157 | TTGACAGAAGATAGAGAGCAC |
| Pau-miR160 | GCGTACGAGGAGCCAAGCATG |
| Pau-miR1 | AGGGCAAGTGGGAGATTGTT |
| Pau-miR26a | TTTCCAACTCCACCCATTCCTA |
