# Supplementary material for: Drought stress-induced changes of microRNAs in diploid and autotetraploid Paulownia tomentosa
Source: Genes Genomics. 2016 Oct 20;39(1):77–86. doi: 10.1007/s13258-016-0473-8 (PMC5196014; doi:10.1007/s13258-016-0473-8)
Supplement: Supplementary file 2 — Supplementary material 2 (DOCX 13 kb) [file 13258_2016_473_MOESM2_ESM.docx]

| Name | Forward sequence (5'-3') | Reverse sequence (5'-3') |
| --- | --- | --- |
| U6 | CTCGCTTCGGCAGCACA | AACGCTTCACGAATTTGCGT |
| CL401.Contig8_All | TCCATTCCGTTACACCATCC | GTCCACTAAGCCAGAACTCC |
| CL10153.Contig2_All | CTGGTCCTGGAGTGTCTG | ACGGATGGCTGAACTATGG |
| CL13082.Contig3_All | CTCGCCAGCAAACTTCAC | GGTATTGTCTCTACAGATTCAGG |
| CL13082.Contig2_All | GCCTGTTGGATGCGGTTC | CTGAATAGCGATGAAGAAGAATGG |
| CL1785.Contig11_All | TCAAGCACCACCACAATCAC | GAAGACAACCGAGACAGGATG |
| CL6480.Contig4_All | ATGGTGTTGATTATTGGATTGTC | CTGGAGAAGGAGGTGATGG |
| Unigene17325_All | TGATAAACTGACTTGATGGGAAAC | ATTGCGAATACGATGATTGACC |
| CL16.Contig8_All | TGAGCGACCGAGTAGACC | GTAGAGACAGTAATATGACCTTGC |

Table S2 Primers of target genes used for qRT-PCR
